# Native Arterio-Venous Fistula Is the Vascular Access of Choice for Hemodialysis in End Stage Renal Disease

**Published:** 2013-06-01

**Authors:** Farooq Ahmad Ganie, Hafeezulla Lone, Abdul Majeed Dar, Ghulam Nabi Lone, Mohd Lateef Wani

**Affiliations:** 1 Department of Cardiovascular and Thoracic Surgery, Soura, Kashmir, India

**Keywords:** Hemodialysis, Chronic Kidney Disease, Arteriovenous Fistula

## Abstract

**Objectives:**

The aim of the study was: Is primary Arterio-venous fistula the vascular access of choice for adequate dialysis and better patient outcome in end stage renal disease.

**Materials and Methods:**

The present study was done in the department of cardiovascular and thoracic surgery at Sher-i-Kashmir institute of medical sciences, Soura, Srinagar Kashmir. Native Arterio-Venous (AV) fistulas were made in the patients with end stage renal disease for performing hemodialysis. They were followed for patency and adequacy of blood flow during hemodialysis. All the patients were operated under local anesthesia.

**Results:**

The results showed that 77% of the AV fistulas based on radial artery with side-to-side anastomosis and 80% of those with end-to-side anastomosis were functionally patent after one year. After two years, the patency rate in side-to-side and end-to-side anastomosis was 50% and 55%, respectively. In addition, the patency rate was 90% in brachial artery based AV fistula with end-to-side anastomosis, whether done primarily or secondarily, at the end of one year. However, a rapid decline was observed in the patency rate during the third year in both radial artery based and brachial artery based AV fistulas.

**Conclusions:**

We concluded that Arterialised arm superficial veins after primary AV fistula was the optimal and rational vascular access for hemodialysis providing adequate blood flow during this process. Besides, failure of primary AV fistula should be replaced by secondary AV fistula preferably based on brachial artery.

## 1. Introduction

Hemodialysis is a mechanical way to cleanse blood and restore body fluid and chemical homeostasis in chronic kidney disease. Hemodialysis is a temporary measure in chronic renal disease in the candidates for renal transplant. Patients with cancer or severe heart disease are not candidates for transplantation and dialysis may be the only option. Hemodialysis needs effective and long lasting vascular access site which can be a native or an arterialised vein (1). Two commonly used vascular accesses are external shunt and the internal Arterio-Venous Fistula (AVF). External shunt is indicated when dialysis must begin immediately and radial artery is mostly used for making the AVF. Anastomosing vein with an artery needs few weeks to mature into a functional fistula. It provides long-lasting access for withdrawing blood during hemodialysis with fewer complications. Immediate thrombosis occurs when endarterectomy is needed before making the AVF. Delayed thrombosis of the AVF occurs by not making use of a proper aseptic technique during cannulation of arterialized vein for hemodialysis. Recurrent blunt trauma and using tight wrist watches or jewelry over the fistula site increase the chances of AVF failure, as well. Phlebitis also causes immediate as well as delayed thrombosis (2).

## 2. Materials and Methods

This retrospective and prospective study was done in the department of cardiovascular and thoracic surgery at Sher-i-Kashmir Institute of Medical Sciences, Soura, Srinagar Kashmir. During the last four years, 177 native AVFs were made in the patients with end stage renal disease for performing hemodialysis. They were followed for patency and adequacy of blood flow during hemodialysis. All the patients were operated under local anesthesia infiltration. Overall, 127, 30, 15, and 5 AVFs were based on left radial artery, right radial artery, left brachial artery, and right brachial artery, respectively. In 131 patients, AVF based on radial artery was made by anastomosing the side of radial artery with the side of cephalic vein (side-to-side anastomosis). On the other hand, AVF based on radial artery was made by anastomosing the proximal end of cephalic vein with the side of radial artery (end-to-side anastomosis) in 26 patients. In 18 patients, brachial artery based AVF was made by anastomosing the distal end of either basillic vein or cephalic vein with the side of brachial artery. Besides, AVF on brachial artery was made by anastomosing the side of cephalic vein with the side of brachial artery in 2 patients. Brachial artery based AVFs were mostly secondary native fistulas for either failure or low blood flow through the primary AVF on radial artery. The arm planned for AVF was prevented from any venous puncture and IV medication for one week before making the AVF. The patients with phlebitis were treated by antibiotics, anti-inflammatory drugs, and heparin ointment for two to three weeks before using the vein for AVF.

### 2.1. Site Determination

The site for AVF was decided by clinical assessment of the veins and flow through radial and ulnar arteries using Allen’s test.

### 2.2. Incision

J-shaped (Ganie incision) and S-shaped incisions were made at the wrist and the elbow, respectively. The length of the incisions varied from 4 to 5 cm. In addition, the longitudinal part of the incision was made on artery and its horizontal part towards the vein.

### 2.3. Technique

Both side-to-side and end-to-side arteriovenous anastomosis were made by continuous suture technique starting from the corner. With 6-zero prolene suture. The Size of fistula which were made was 1 to 1.8cms on radial artery and 8 to 10mm on brachial artery. Excessive electro-cautery was avoided to prevent nerve damage. Moreover, all the patients were advised to keep the arm with AVF elevated by 20 to 30 degrees for 5 to 7 days.

## 3. Results

The study results showed that 77% of AVFs based on radial artery with side-to-side anastomosis and 80% of those with end-to-side anastomosis were functionally patent after one year. After two years, the patency rate in side-to-side and end-to-side anastomosis was 50% and 55%, respectively. In addition, the patency rate was 90% in brachial artery based AVFs with end-to-side anastomosis, whether done primarily or secondarily, at the end of one year. However, a rapid decline was detected in the patency rate during the third year in both radial artery based and brachial artery based AVFs. The functional patency of AVF with a flowing capacity of more than 200mL/min during hemodialysis was correlated with an audible murmur at a distance over 6 cm from the fistula site by hand held vascular Doppler. In comparison to the AVFs made by side-to-side anastomosis, those made by end-to-side anastomosis had comparatively more blood flow during hemodialysis possibly due to bidirectional flow in side-to-side anastomosis. Nonetheless, more arterialized veins were available for cannulation during hemodialysis when AVF was made by side-to-side anastomosis on radial artery. In this study, 2 patients with brachial artery based AVF needed reduction of the fistula size in view of arm edema. Also, 3 patients with severely atherosclerotic radial artery needed endarterectomy before anastomosis. However, none of the patients developed early thrombosis. Ten patients with radial artery based AVF complained about minor parasthesia over the thenar eminence. Overall, brachial artery based side-to-side AVF provided less blood flow during hemodialysis due to more run-offs towards the central circulation.

### 3.1. Failure and Management

Immediate thrombosis and blockage was managed by re-fashioning the blocked AVF by end-to-side anastomosis. Delayed blockage was replaced by secondary AVF on brachial artery by end-to-side anastomosis. It was technically easier to make secondary native AVF on the same arm at a different site due to venous dilatation by the previous AVF.

## 4. Discussion

Native AVF is exclusively used for hemodialysis not only for limited resources, but also for optimal functional results. As per Dialysis Outcomes and Practice Patterns Study (DOPPS), native AVF is the vascular access of choice for adequate dialysis and better patient outcome. However, graft is a better alternative than catheter for the patients for whom creation of an AVF has failed (1). The most frequently observed complications include aneurysm (51%) followed by venous hypertension (16.7%), infection (4.4%), thrombosis (3.3%), and arterial steal syndrome (1.1%) (2). In the current study, venous hypertension and arm edema were the most prevalent complications followed by thrombosis of the AVF. Edema was seen mostly after brachial artery based end-to-side AVF, but this edema regressed spontaneously in the majority of the patients, except for two patients who needed narrowing of the AVF. Aneurysm formation in relation to AVF was seen in only two patients. Besides, arterial steal syndrome was detected in two patients with brachial artery based AVF with side-to-side anastomosis. In general, cannulation of AVF is technically more challenging than that of AV grafts (3). After 4 to 6 weeks of maturation, cannulating an arterialized vein after AVF formation is not difficult. Stenosis is considered as the major cause of AVF dysfunction (4). In this study, thrombosis was revealed as an important cause of malfunction in immediate follow-up. Among AVF, AV graft, and central venous catheter, AVF is the ideal choice of vascular access (5). After native AVF, arterialized vein was observed to be a better choice for easy vascular access, long-lasting functional patency, and optimal blood flow during hemodialysis. Previously used veins for primary AVF are excellent candidates for creation of secondary AVF (6). Moreover, after decreased flow or failure of the primary native AVF, the secondary native AVF has optimal functional patency when made on brachial artery. Secondary native AVF was technically easy to make due to previous venous dilatation. First cannulation of AVF varies significantly between countries: Japan, 25 days; Italy, 27 days; Germany, 42 days; Spain, 80 days; France, 86 days; United Kingdom, 96 days; and the United States, 98 days (7). Native AVF is used after 4 to 6 weeks with optimal patency and functional results. Placement of upper extremity grafts often results in thrombosis or stenosis of the upper arm superficial and deep veins, making future placement of a native fistula more demanding (8). Native AVF is an optimal method for vascular access whether it is primary or secondary. In the present study, only 8 patients developed thrombosis of arterialized superficial venous channels after a two-year follow-up. The main cause of this delayed thrombosis was the improper technique of cannulation, not the creation of native AVF. In general, creation of an AVF increases the left ventricular volume in renal transplant patients (9). In this study, one patient had a clinically significant volume load of the left ventricle with brachial artery based side-to-side AVF. Although the central venous catheter is the vascular access of last choice, it can be a useful alternative in particular cases, (10). We observed that the central venous catheter was a temporary vascular access until the patients received native AVF. Also, it was observed that central venous catheter management needs more expertise and strict patient cooperation as well as self-care and creates psychosocial apprehensions. Thus, other vascular accesses should be used only when all the options of native AVF are exhausted. Primary AVF patency was shorter in chronic renal insufficiency with diabetes mellitus, malignancy, and previous catheter insertion, while longer in the patients using heparin for hemodialysis and hemodialysis count per week (>3) (11). We determine that hyperlipideamia, uncontrolled diabetes, hypertension, and improper control of renal parameters by inadequate hemodialysis were the risk factors for the functional patency of native AVF. The rate of morbidity and mortality is lower in the patients with native fistula compared to those with permanent catheters or fistulas made of artificial materials (12). The clinically significant morbidities investigated in the current study were arm edema due to venous hypertension and patency failure requiring re-fashioning of the native fistula. The available techniques for creating Brachio-Basilic AVF (BBAVF) are associated with a good patency rate and most related complications can be treated conservatively without loss of the fistula (13). An upper arm AVF is a reasonable alternative for maintenance of hemodialysis access when a radio-cephalic AVF is not possible (14). Brachial artery based AVF was made mostly as a secondary fistula for failure of the primary radio-cephalic AVF and its primary patency, functional patency, and durability were also comparable to primary native AVF. BBAVF and brachio-cephalic AVF are equally effective alternatives; however, a longer and more demanding operation with BBAVF construction should be considered (15).

The findings of the present study revealed no significant difference in duration of making fistula based on brachial or radial artery; nevertheless, patient compliance with brachial artery dissection was unacceptable. Moreover, brachio-cephalic fistulas were superior to radio-cephalic regarding their maturation rate, and functional patency (16).

However, radial artery based side-to-side AVF is technically easier to make with better patient compliance. Of course, dissecting brachial artery under local anaesthesia is sometimes uncomfortable for the patients.

## 5. Conclusion

Arterialised arm superficial veins after native AVF, whether primary or secondary, proved to be the optimal and rational vascular access for hemodialysis providing adequate blood flow during the process. Failure of primary native AVF should be replaced by native secondary AVF preferably based on brachial artery. However, further studies are needed to establish the facts. Cannulation of the arterialized vein should be done at a distance of more than 10 cm from the AVF site to prevent thrombosis of the fistula.

## References

[1] Ethier J, Mendelssohn DC, Elder SJ, Hasegawa T, Akizawa T, Akiba T (2008). Vascular access use and outcomes: an international perspective from the Dialysis Outcomes and Practice Patterns Study.. Nephrol Dial Transplant..

[2] Derakhshanfar A, Gholyaf M, Niayesh A, Bahiraii S (2009). Assessment of frequency of complications of arterio venous fistula in patients on dialysis: a two-year single center study from Iran.. Saudi J Kidney Dis Transpl..

[3] Ball LK (2005). Improving arteriovenous fistula cannulation skills.. Nephrol Nurs J..

[4] Campos RP, Do Nascimento MM, Chula DC, Do Nascimento DE, Riella MC (2006). Stenosis in hemodialysis arteriovenous fistula: evaluation and treatment.. Hemodial Int..

[5] Murphy F (2011). The ongoing challenges with renal vascular access.. Br J Nurs..

[6] Asif A (2007.). Secondary Arteriovenous Fistulae..

[7] Rayner HC, Pisoni RL, Gillespie BW, Goodkin DA, Akiba T, Akizawa T (2003). Creation, cannulation and survival of arteriovenous fistulae: data from the Dialysis Outcomes and Practice Patterns Study.. Kidney Int..

[8] Ascher E, Hingoran A, Gunduz Y, Yorkovich Y, Ward M, Miranda J (2001). The value and limitations of the arm cephalic and basilic vein for arteriovenous access.. Ann Vasc Surg..

[9] Iwashima Y, Horio T, Takami Y, Inenaga T, Nishikimi T, Takishita S (2002). Effects of the creation of arteriovenous fistula for hemodialysis on cardiac function and natriuretic peptide levels in CRF.. Am J Kidney Dis..

[10] Quarello F, Forneris G, Borca M, Pozzato M (2006). Do central venous catheters have advantages over arteriovenous fistulas or grafts?. J Nephrol..

[11] Erkut B, Unlu Y, Ceviz M, Becit N, Ates A, Colak A (2006). Primary arteriovenous fistulas in the forearm for hemodialysis: effect of miscellaneous factors in fistula patency.. Ren Fail..

[12] Weyde W, Krajewska M, Klinger M (2006). [Vascular access for hemodialysis in difficult patients--procedure principles based on own experiences].. Pol Merkur Lekarski..

[13] Hossny A (2003). Brachiobasilic arteriovenous fistula: different surgical techniques and their effects on fistula patency and dialysis-related complications.. J Vasc Surg..

[14] Fitzgerald JT, Schanzer A, Chin AI, McVicar JP, Perez RV, Troppmann C (2004). Outcomes of upper arm arteriovenous fistulas for maintenance hemodialysis access.. Arch Surg..

[15] Koksoy C, Demirci RK, Balci D, Solak T, Kose SK (2009). Brachiobasilic versus brachiocephalic arteriovenous fistula: a prospective randomized study.. J Vasc Surg..

[16] Nguyen TH, Bui TD, Gordon IL, Wilson SE (2007). Functional patency of autogenous AV fistulas for hemodialysis.. J Vasc Access..

